# The Linguistics of Hypertension: Is “Essential” Really Primary, or Just Plain Complex?

**DOI:** 10.1038/s41371-025-01066-w

**Published:** 2025-09-10

**Authors:** Victoria D. Dahmen

**Affiliations:** Division of Experimental Medicine, University of Cambridge, Addenbrooke’s Hospital, Cambridge, CB2 0QQ UK

**Keywords:** Cardiovascular biology, Hypertension

## Abstract

The Stanley Peart Essay Competition is an annual event run by the British and Irish Hypertension Society to encourage Early Career Researchers to continue the ethos of Sir Stanley Peart. Sir Stanley Peart was a clinician and clinical researcher who made a major contribution to our understanding of blood pressure regulation. He was the first to demonstrate the release of noradrenaline in response to sympathetic nerve stimulation. He was also the first to purify, and determine the structure of, angiotensin and he later isolated the enzyme, renin, and carried out many important investigations of the factors controlling its release in the body. This year, the essay topic was “Is Essential Hypertension really Primary or Essential?”. In her prize-winning essay, Victoria Dahmen questions the continued use of the term “essential hypertension” suggesting that it may obscure the complex and often modifiable factors contributing to elevated blood pressure. By exploring the roles of salt sensitivity, obesity, and their physiological interplay, her essay calls for a more nuanced, mechanism-based classification of hypertension. Such a shift, she argues, will facilitate clinicians to deliver personalised medicine by aligning medical language with modern scientific understanding.

## “Essential”: A Linguistic Journey

The lexicon of medicine, much like any language, evolves to categorise and communicate understanding. The term “primary hypertension” currently functions as a diagnosis of exclusion. If no secondary causes, such as renal disease, pheochromocytoma, aldosteronism, or monogenic hypertension, are present [1], a clinician will default to this fundamental label. Labelling hypertension as “essential” only after ruling out known secondary causes may delay more detailed investigation into less obvious contributors. For instance, mild aldosteronism, often missed on routine examination, can present as “essential” hypertension but responds to targeted treatment. Similarly, undiagnosed pheochromocytoma, paraganglioma, renal artery stenosis or aortic coarctation may hide under the same label. This raises an important concern: does the term “essential” obscure potentially modifiable causes by implying that the condition is inherently unexplainable? And more importantly: how did we get there?

The term “essential hypertension” was coined at the beginning of the 20th century to imply that increasing blood pressure arises as the body’s natural response to arteriolar constriction to prevent ischaemia [2]. Since then, science has moved on, yet the misnomer remained in medical textbooks. Eventually, evidence began accumulating for risk factors of “essential” hypertension, including obesity, stress and salt intake. In the 1930s, newly immigrated to North Carolina German physician Walter Kempner proposed a dietary intervention to unburden the kidneys of excess pressure - a rice diet composed of carbohydrates with minimal amounts of salt and protein [3]. Perhaps through a misunderstanding of his instructions due to the particularly heavy German accent, a hypertensive patient adhered to the diet for two months instead of the intended two weeks, yielding unprecedented reductions in blood pressure [4]. Advantages of Kempner’s rice diet marked a paradigm shift in the field of hypertension: was salt the cause of and salt reduction the cure for so-called essential hypertension? Further, even if salt is not the cause, does essential hypertension truly denote an idiopathic phenomenon, or merely mask a complex cause?

In this essay, I will argue that the phrase “essential hypertension” is semantically misleading, as it fails to capture the multifactorial reality of the hypertensive syndrome. Instead, this idiopathic hypertension is predominantly driven by the semantic weight of obesity, the physiological language of salt overload and the dialect of interaction between the two.

## Unpacking Salt’s Story

The linguistic categorisation of patients as “salt-sensitive” or “salt-resistant” underscores the inherent variability in physiological responses, a nuance often obscured by the broad term “primary hypertension”. According to Arthur Guyton’s model of salt handling, a dominance of salt intake over renal excretion initiates a cascade of increased extracellular fluid osmolality, blood volume expansion, elevated cardiac output and a resultant rise in arterial blood pressure [5]. The counter-regulatory mechanism of pressure natriuresis, where heightened glomerular filtration pressure proportional to arterial pressure drives increased sodium excretion, ideally restores long-term sodium balance. In Guyton’s framework, the very definition of “salt sensitivity” hinges on a compromised capacity of the kidneys to appropriately modulate this pressure-natriuresis curve in response to sodium imbalances.

The International Study of electrolyte excretion and blood pressure (Intersalt) [6] sought to empirically validate Guyton’s predictions by quantifying the relationship between urinary electrolyte excretion and blood pressure, revealing a linear association between urinary Na^+^ excretion and systolic blood pressure [6]. This finding provided a foundation for testing the hypothesis that a dietary intervention of reduced sodium intake could lead to a fall in blood pressure, a concept later supported by a meta-analysis of 34 trials [7]. However, the inherent averaging within such meta-analyses can obscure the individual categories of salt-sensitive and salt-insensitive responses observed in hypertensive subgroups challenged with high-sodium diets. Intriguingly, the mechanisms underlying salt sensitivity, at least in black individuals, appear to be driven not primarily by renal function but by the pressor effect of vascular dysfunction [8]. This suggests that the semantic domain of “salt sensitivity” may encompass diverse underlying physiological mechanisms.

Yet, the “salt story”, to continue our linguistic analogy, presents further complexities that extend beyond the kidney’s immediate lexical field. The observation that most individuals, even those with hypertension, are not salt sensitive prompts the question: what physiological grammar underlies this resistance to the blood-pressure-raising effects of salt?

Emerging research into non-osmotic sodium storage in the skin [9], mediated by the polymerization of negatively charged glycosaminoglycans attracting positively charged Na^+^ [10], offers a potential linguistic bridge to explain this variability. This skin storage could act as a buffer, modulating the impact of excess salt in salt-resistant individuals [9], and alterations in this buffering capacity might contribute to the development of salt-sensitive hypertension. The observed sexual dimorphism in this skin-buffering capacity offers a potentially semantic link to the differential prevalence of hypertension between men and women [11], although further research is needed to solidify this connection. Could we, therefore, refine our diagnostic lexicon to categorise individuals based on this buffering capacity?

The evidence for the role of dietary salt in hypertension development and salt reduction in hypertension treatment is substantial, as acknowledged by the European Society of Hypertension [12]. The salt story offers an opportunity for the hypertension community to introduce a mechanistically informed language of hypertension. The current practice of grouping diverse individuals under the umbrella term “primary hypertension” may lead clinical trials to overlook the efficacy of drugs that specifically target the underlying mechanisms in subpopulations, such as those with salt-sensitive hypertension. Understanding the underlying mechanism, whether rooted in renal syntax or the emerging semantics of extra-renal sodium handling, is essential for developing truly effective and personalised anti-hypertensive therapies.

## Weighty Words, Complex Links

The frequent co-occurrence of the lexical items “obesity” and “hypertension” in medical literature points to a strong associative link, almost as if “obesity-related hypertension” were a more descriptive, albeit longer, diagnostic phrase. Time and again, the data reveals a significant correlation: excess weight is associated with an increased risk of hypertension [13, 14], with prevalence nearly doubling in overweight patients and exceeding 70% in those with obesity [15]. More convincingly, gaining weight is associated with an increased risk of developing hypertension [16], while losing weight has the opposite effect [17], suggesting a far more direct and actionable relationship than “essential” implies. The continued reliance on “essential hypertension” risks obscuring the substantial contribution of obesity to hypertension.

The challenge lies in ensuring our medical lexicon accurately reflects the underlying pathophysiology, avoiding semantic shortcuts that might imply false causality or misrepresent the complex interplay of variables. The mechanisms of what we might more accurately term ‘obesity-mediated hypertension’ involve a complex physiological language: overactivation of the renin-angiotensin-aldosterone system, chronic sympathetic overactivation, and the physical compression of the kidney by visceral fat [18, 19], all contributing to a disruption in the kidney’s own regulatory discourse of pressure natriuresis. The long-term consequences, including dyslipidaemia and type-2 diabetes leading to chronic kidney disease [18], further complicate the semantic field surrounding hypertension, highlighting a web of interconnected conditions rather than a singular, unexplained entity.

The bidirectional relationship - lean hypertensive patients being more prone to developing obesity [20, 21] - further complicates any simplistic causal narrative. Weight loss demonstrably reduces hypertension; however, the pre-existing blood pressure affects the final pressure achieved with weight loss [22]. In addition, established hypertensives have a harder time losing weight [23, 24], suggesting a more intricate semantic entanglement than a straightforward cause-and-effect relationship. One potential explanation is that hypertension driven by sustained increases in sympathetic output leads to downregulated responsiveness of beta-adrenergic receptors, driving insufficient thermogenesis, thereby contributing to weight gain [25]. Alternatively, there is a confounding factor, e.g., a common genetic background predisposing the population to both hypertension and obesity.

The observation that hypertension might not be solely “obesity-driven” but rather linked to components associated with obesity [21] underscores the need for more precise terminology. Caloric restriction and the accompanying weight loss were shown to reduce sympathetic activity in obese subjects [26], which can be due to a multitude of factors, including improvement in sympathetic responsiveness, lowering leptin concentration or restored insulin sensitivity. To complicate matters further, defects in beta-adrenergic sensitivity of a lymphocyte model of beta-adrenoreceptor regulation, can be corrected with low sodium diet [27], further highlighting the interconnectedness often lost under the broad rubric of “primary”.

Recognising the semantic weight of obesity in hypertension necessitates a move away from a label like “essential”, which fails to capture this critical and, now more than ever, modifiable contributing factor. We are doing patients a disservice by using “essential” as a euphemism for complex and difficult to treat. “Essential” might be misinterpreted by patients as meaning the condition is somehow unavoidable, even though it is not.

## Complex Problems, Simple Solutions?

Pharmacological weight loss with the use of glucagon-like peptide-1 receptor agonists contribute to reduction in blood pressure [28], at least partially through reduction in body weight. Unexpectedly, GLP-1R antagonism was found to lower pressure natriuresis in a spontaneously hypertensive rat model [29], implicating the role of endogenous GLP-1 in natriuresis regulation. Perhaps, not only the causes of obesity-associated hypertension, but also the mechanisms underlying their solutions are more interconnected than one would predict.

Similarly, the historical success of Kempner’s rice water diet, which targeted two major contributors to hypertension by simultaneously addressing salt intake and weight, underscores the interconnectedness of these contributors. It also provides an example, where a simple strategy can be used to successfully manage the complexity of hypertension.

## Towards A Clearer Nomenclature

The evidence presented above leads me to put forward that the essence of essential hypertension lies not in its “primary” nature but in its pathophysiological complexity. Primary hypertension is not really a single disease, but rather a syndrome, a final common pathway of diverse mechanisms. Rather than following a clear timeline and a string of causal events, hypertension arises from the interplay of various factors, including salt sensitivity, obesity and sympathetic overdrive, as discussed.

So, where does this leave us? Perhaps it’s time to abandon the misleading labels altogether. Instead of “essential” or “primary”, should we embrace a more nuanced, systems-based approach? Imagine a classification that acknowledges the multifaceted nature of the disease, categorising patients based on their dominant pathophysiological drivers: “salt-sensitive hypertension”, “sympathetic-driven hypertension” etc.

Scientists in the field of hypertension appreciate the complexity hidden under the innocent words “primary” or “essential”, but the wider public would benefit from a greater understanding of the underlying, largely modifiable causes. We should stop hiding behind euphemisms, as the potential advantages of changing the nomenclature vastly outweigh the transient discomfort of a mouthful. Such a naming system would be more scientifically accurate, necessitate a better patient stratification in clinical trials and offer a more personalised approach to diagnostics and treatment. Imagine a future in which we could routinely test for salt-sensitivity of patients, and tailor the therapy to their needs. For the salt-sensitive patient, a low-sodium diet, diuretics and potentially novel drugs increasing the buffer capacity of skin; for the obesity-associated patient, weight-loss drugs and therapies modulating the contribution of the sympathetic nervous system.

## The End of An “Essential” Era

In conclusion, the term “essential hypertension” is a historical relic, a testament to our past ignorance. Both “primary” and “essential” fail to capture the intricate complexity of the condition. Our understanding of hypertension has evolved massively since the beginning 20th century, and so should our language.

Is it better to subdivide the current primary hypertension diagnosis into multiple different subcategories, a scale with contributions of individual risk factors, or continue the research trajectory aimed at identifying distinct etiological pathways within what we currently call primary hypertension? This remains to be discussed. Regardless, “primary” must go!

## Data Availability

No data was generated for the development of this manuscript.
